# Development and Application of Patient-Reported Outcome Measures (PROMs) in Patients on Chronic Home Oxygen Therapy

**DOI:** 10.3390/jcm15134948

**Published:** 2026-06-25

**Authors:** Eusebi Chiner, Ignacio Boira, Joaquín Fernández-Serrano, Mónica Llombart, Violeta Esteban, Paula Fernández Martínez, Marian Fernández, Sandra Vañes, Francesco Gigliarano, Sandra Navarro, Sergio García Ferrer

**Affiliations:** 1 Pulmonology Department, San Juan de Alicante University Hospital, 03550 Sant Joan d’Alacant, Alicante, Spain; echinervives@gmail.com (E.C.); fernandez.joaquin@umh.es (J.F.-S.); llombartcanto@gmail.com (M.L.); violeta_er@hotmail.com (V.E.); paulafernandez1441@gmail.com (P.F.M.); 2 Faculty of Medicine, Universidad Miguel Hernández, 03550 Sant Joan d’Alacant, Alicante, Spain; 3 Linde Healthcare, 28108 Alcobendas, Madrid, Spain; marian.fernandez@linde.com (M.F.); sandravanesbanos@gmail.com (S.V.); francesco.gigliarano@linde.com (F.G.); sandra.navarro@linde.com (S.N.); sergio.garcia.ferrer@linde.com (S.G.F.)

**Keywords:** chronic home oxygen therapy, long-term oxygen therapy, LTOT, patient-reported outcome measures, PROMs, chronic respiratory failure, COPD, adherence, quality of life, validation study

## Abstract

**Background/Objectives**: Chronic home oxygen therapy—long-term oxygen therapy (LTOT)—improves survival and quality of life in chronic respiratory failure when used ≥15 h/day, but adherence is frequently suboptimal and specific patient-reported outcome measures (PROMs) are scarce. To develop, validate and apply a specific PROM for patients on LTOT. **Methods**: A prospective observational cohort study was conducted at San Juan de Alicante University Hospital (April 2024–December 2025) following a four-stage process: conceptual framework definition and expert workshop, content validation and item reduction, cognitive interviews with pilot reliability testing (n = 25), and field application to 120 consecutive chronic LTOT users. The LTOT-PROM was designed to capture the patient-perceived impact attributable to LTOT during the previous 4 weeks. Internal consistency was assessed with Cronbach’s α and test–retest reproducibility with the intraclass correlation coefficient (ICC). **Results**: The final instrument comprises 15 scored items in two dimensions—Daily Activity (9 items) and Adverse Effects (6 items)—plus one ambulatory-only mobility item excluded from the total score. Cronbach’s α was 0.814 (95% CI 0.681–0.906) for Daily Activity, 0.743 (95% CI 0.548–0.872) for Adverse Effects and 0.808 (95% CI 0.677–0.902) for the total scale; total ICC(A,1) was 0.890 (95% CI 0.767–0.950). Among the 120 patients (62 men, 58 women; mean age 78 ± 13 years; mean therapy duration 40 ± 32 months), 68% reported reduced effort for daily activities, 66% reported a reduction in dyspnoea and 67% reported improved self-confidence; 49% reported morning airway dryness and 7% abandoned the equipment due to nasal dryness or rhinitis. **Conclusions**: The LTOT-PROM is a brief, reliable and reproducible oxygen-specific instrument for assessing the recent patient-perceived impact of LTOT in routine clinical practice. Further studies should evaluate structural validity, external validity and the relationship between LTOT-PROM scores and objective adherence measures. The construct was predefined as the patient-perceived impact attributable to LTOT during a standardised 4-week recall window, and cognitive interviews confirmed that respondents interpreted the items as experienced benefit/burden during that period rather than as week-to-week symptom change.

## 1. Introduction

Chronic respiratory failure (CRF) is a syndrome in which the respiratory system fails in one or both of its gas-exchange functions, leading to arterial hypoxaemia (PaO_2_ < 60 mmHg) with or without hypercapnia, and to a wide spectrum of clinical consequences including pulmonary hypertension, cor pulmonale, secondary polycythaemia, exercise intolerance, neurocognitive impairment and progressive loss of autonomy [1]. CRF represents the final pathway of many advanced respiratory and cardiac diseases—most prominently chronic obstructive pulmonary disease (COPD), interstitial lung disease, neuromuscular disorders and chronic heart failure—and is a major source of morbidity, mortality and healthcare resource use in ageing populations. COPD alone is currently estimated to be the third leading cause of death worldwide, with a rising prevalence driven by demographic ageing, persistent tobacco exposure and environmental pollution, and remains the archetypal indication for long-term oxygen therapy in clinical practice [2].

Long-term oxygen therapy (LTOT) is a cornerstone treatment for chronic severe hypoxaemia when the underlying mechanism is expected to improve with oxygen supplementation. In disorders dominated by ventilation–perfusion mismatch or diffusion impairment—particularly COPD and many fibrotic or vascular interstitial lung diseases—LTOT is generally beneficial when the guideline criteria are met. By contrast, in hypoventilation syndromes such as obesity hypoventilation syndrome or some neuromuscular disorders, ventilatory support is often the primary long-term treatment and may correct hypoxaemia without isolated oxygen therapy. Likewise, in patients with hypercapnia, oxygen treatment may need to be integrated with ventilatory support and careful reassessment. LTOT should therefore be interpreted as a pathophysiologically targeted therapy rather than as a uniform treatment for every form of chronic respiratory failure [3,4,5,6,7,8,9,10].

The survival benefit of LTOT in severe COPD was originally established more than four decades ago by the British Medical Research Council (MRC) trial [3] and the North American Nocturnal Oxygen Therapy Trial (NOTT) [4], whose combined results showed that continuous oxygen use (≥15 h/day) reduced all-cause mortality compared with nocturnal-only or no oxygen. These landmark studies, together with subsequent systematic reviews [5], shaped clinical practice worldwide and established the fundamental dose–response concept that survival benefit is proportional to daily hours of use.

Over the past decade, however, the indications and expected benefits of LTOT have been re-examined. The Long-Term Oxygen Treatment Trial (LOTT) demonstrated that, in patients with stable COPD and moderate resting desaturation (SpO_2_ 89–93%) or exercise-induced desaturation, supplemental oxygen did not prolong the time to death or first hospitalisation [6]. More recently, the multicentre INOX trial showed no significant survival or hospitalisation benefit from isolated nocturnal oxygen in COPD patients with nocturnal arterial desaturation but without daytime severe hypoxaemia [7]. Together with modern observational evidence [8], these data have refined the clinical scope of LTOT: the consensus today is that the therapy is unambiguously indicated for patients with severe resting hypoxaemia (PaO_2_ ≤ 55 mmHg, or ≤60 mmHg in the presence of cor pulmonale or polycythaemia), where it improves not only survival but also exertional dyspnoea, sleep quality and health-related quality of life [9].

Indications for LTOT have therefore been progressively standardised across high-level international and national guidelines. The American Thoracic Society’s (ATS’s) clinical practice guideline on home oxygen therapy in adults with chronic lung disease provides graded recommendations for COPD and interstitial lung disease, and explicitly recognises both the survival and symptomatic dimensions of the therapy, as well as the educational and equipment-related challenges encountered in real-world practice [10]. National guidance documents from Spain [11,12,13], the United Kingdom [14,15] and other countries have aligned with this evidence base, while recognising the substantial heterogeneity in how oxygen is prescribed, monitored and reassessed. Home oxygen can be delivered by stationary concentrators, liquid oxygen reservoirs or portable (ambulatory) systems, with the latter being increasingly used to preserve patient mobility and social participation. The economic and organisational impact of home respiratory therapies is substantial: they represent one of the largest items of expenditure within home-based chronic care, with continuous growth driven by population ageing and the rising prevalence of chronic respiratory disease [16,17].

Despite this robust evidence base and continuous refinement of indications, adherence to LTOT remains a major unmet need. Real-world studies consistently show that fewer than half of patients reach the recommended daily use of ≥15 h/day, with a substantial proportion using oxygen for less than 12 h per day [18]. Reported barriers to adherence include nasal dryness and rhinitis, social stigma associated with visible equipment, fear of becoming dependent on oxygen, equipment-related discomfort, active smoking and limited disease literacy [18,19,20]. Among these, mucosal and airway dryness deserves particular attention, as it is one of the most frequent symptomatic complaints and a recognised cause of discontinuation; the role of the routine humidification of low-flow-inspired oxygen, however, remains controversial, with bench and clinical data showing only modest benefit and considerable inter-individual variability [21]. Qualitative research has further highlighted that patients and caregivers experience LTOT as a profound biographical disruption, with implications for personal identity, family dynamics and social interactions that are seldom captured by standard clinical metrics [22,23].

Within this context, Patient-Reported Outcome Measures (PROMs) have emerged as a key methodological development to reorient chronic disease management toward genuinely patient-centred care. PROMs are standardised, validated instruments that capture the patient’s own perception of their health status, symptoms, functional capacity and treatment effects, without interpretation by clinicians or third parties [24,25]. Over the last two decades, their systematic incorporation into routine practice, clinical trials and quality-improvement programmes has been advocated as one of the most promising strategies to improve the quality, transparency and value of healthcare [26,27,28]. Beyond research, PROMs can be used at the individual level to support shared decision-making, identify unmet needs and tailor self-management interventions, and at the population level as quality indicators for benchmarking services and evaluating new models of care [27,28,29]. Recent international initiatives, including the SPIRIT-PRO and CONSORT-PRO extensions, have additionally established methodological standards for the design, reporting and analysis of PRO data in clinical research [28,29].

Generic respiratory PROMs—most notably the St George’s Respiratory Questionnaire (SGRQ) [30]—have been extensively used in COPD and other chronic lung diseases. However, they were designed to capture the global impact of respiratory disease and not the specific experience of being on home oxygen therapy. As a consequence, key aspects such as equipment comfort, social interference, fear of dependence, mucosal dryness or perceived mobility gains are either absent or only marginally represented. To the best of our knowledge, the only validated Spanish initiative addressing PROMs and Patient-Reported Experience Measures (PREMs) across the broader field of home respiratory therapies (oxygen, CPAP, mechanical ventilation and aerosol therapy) is the work of Rudilla et al. [31]; while methodologically sound, the resulting instrument is comparatively long and not oxygen-specific, which limits its routine application in busy outpatient pulmonology clinics. Patient-centred clinical-trial designs in COPD have also highlighted the value of involving patients directly in the conception of adherence-promoting interventions for supplemental oxygen [32], underscoring the need for short, condition-specific, patient-friendly tools that can be deployed in everyday respiratory care.

We therefore hypothesised that a brief, specific and validated PROM for LTOT—structured around the two domains most relevant to patients, namely perceived therapeutic benefit in daily life and perceived adverse effects of the therapy—would be feasible in routine outpatient pulmonology practice and show adequate internal consistency and test–retest reproducibility. The LTOT-PROM was conceived as an anchored measure of the patient-perceived impact attributable to LTOT during the previous 4 weeks in chronic users.

Accordingly, the primary objective of this study was to develop a specific PROM for patients on LTOT. Secondary objectives were: (i) to test its reliability and reproducibility; and (ii) to apply it to a real-world cohort in order to evaluate therapeutic response and adverse effects.

## 2. Materials and Methods

### 2.1. Study Design and Setting

A prospective, observational cohort study was carried out between April 2024 and December 2025.

### 2.2. Ethics Statement

The study protocol was approved by the Ethics Committee for Research with Medicines (CEIm) of San Juan de Alicante University Hospital (reference code 23/075) and was conducted in accordance with the Declaration of Helsinki. All participants provided written informed consent.

### 2.3. Study Population

Consecutive adult patients (≥18 years) on stable LTOT—either stationary (concentrator/liquid oxygen at home) or ambulatory (portable systems)—prescribed for at least three months and able to read and understand Spanish were included. Patients with cognitive impairment preventing self-completion or in palliative end-of-life situations were excluded.

In our centre, LTOT is prescribed in routine practice by pulmonologists for patients with chronic hypoxaemic respiratory failure who meet the accepted clinical indication criteria. Accordingly, the study population corresponds to patients with established home-oxygen requirements, even though formal physiological severity descriptors were not systematically collected for this initial development study.

### 2.4. PROM Development Process

**Figure 1:**
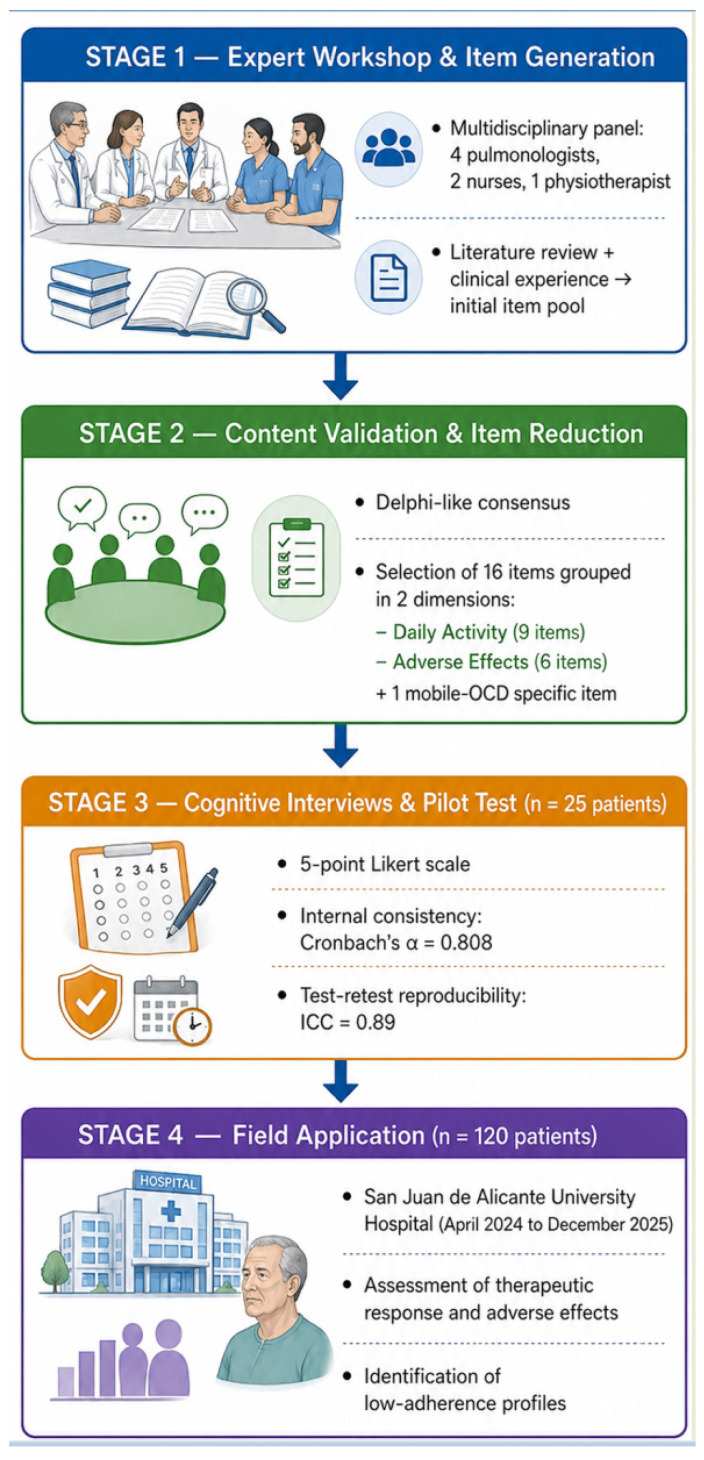
Four-stage process used to develop the instrument.

The instrument was developed following a four-stage process (Figure 1).

Before pilot testing, the target construct was explicitly defined as the patient-perceived impact attributable to LTOT over the last 4 weeks in two domains: (1) perceived therapeutic benefit in daily activity and (2) treatment-related adverse effects. Thus, the 4-week frame was used as a standardised recall window for current lived experience while receiving LTOT, not as a requirement that symptoms must have changed between week 1 and week 4.

Stage 1—Conceptual Framework Definition and Expert Workshop: Before drafting the items, the research team specified the construct of interest as the patient-perceived impact attributable to LTOT during the previous 4 weeks in chronic users. A multidisciplinary panel composed of four pulmonologists, two specialised nurses and one respiratory physiotherapist then generated a pool of candidate items through brainstorming sessions, a review of the published literature [9,10,13,22,23,30,31] and everyday clinical experience. This stage focused on identifying the dimensions most relevant to patients receiving chronic home oxygen therapy, particularly perceived benefit in daily life and therapy-related burden.

Stage 2—Content Validation and Item Reduction: The initial item pool underwent a Delphi-like consensus process in which the experts assessed conceptual relevance, clarity, redundancy and suitability for routine outpatient use. Items with overlapping meaning were merged or removed, and the remaining items were grouped into two conceptual dimensions—Daily Activity and Adverse Effects. Particular attention was paid to maintaining simple wording, direct clinical interpretability and a completion time compatible with daily practice. A 5-point Likert response scale was adopted (“nothing”, “very little”, “somewhat”, “quite a lot”, “a lot”).

Stage 3—Cognitive Interviews and Pilot Reliability Testing: The preliminary questionnaire was administered to 25 patients on LTOT. Comprehensibility, acceptability and item interpretation were explored by structured cognitive interview, with special attention to whether patients understood the common time anchor (“in the last 4 weeks”) and interpreted the comparative verbs consistently with recent LTOT-related experience. The questionnaire was re-administered 7–14 days later in clinically stable patients to estimate test–retest reproducibility.

During the cognitive interviews, patients were specifically instructed to answer each item according to the extent to which oxygen therapy had made breathing, effort tolerance, daily functioning or treatment burden easier/better (or more troublesome, for adverse effects) during the previous 4 weeks. In other words, the instrument was designed as an attribution-based PROM for routine follow-up, rather than as a pure transition scale measuring short-term within-month change.

Stage 4—Field Application: The final instrument comprised 15 scored items in two dimensions—Daily Activity (9 items) and Adverse Effects (6 items)—plus one ambulatory-only descriptive item on mobility (item 2b), which was retained because of its clinical relevance but excluded from the total score. The final questionnaire was applied to 120 consecutive patients on LTOT in order to evaluate perceived therapeutic response and adverse effects under real-world conditions.

### 2.5. Variables

Demographic and clinical variables included age, sex, underlying disease, indication for LTOT, daily hours of use and total months on therapy. PROM responses were coded from 0 (“nothing”) to 4 (“a lot”). For dimension scoring, items reflecting benefit in Daily Activity were considered positive when answered “somewhat”, “quite a lot” or “a lot”, whereas in Adverse Effects the absence of the adverse event (“nothing” or “very little”) was considered the favourable outcome. For total-score interpretation, adverse-effect items were reverse-coded so that higher values reflected a more favourable overall LTOT experience. Item 2b was analysed descriptively and excluded from the total score. Formal physiological severity markers (e.g., arterial blood gases, FEV1, mMRC or 6 min walk distance) were not consistently available in the clinical database and were therefore not prespecified in this development study.

### 2.6. Statistical Analysis

Quantitative variables are expressed as mean ± standard deviation (SD) or median (interquartile range); qualitative variables as absolute frequencies and percentages. Internal consistency was assessed with Cronbach’s α (α ≥ 0.70 acceptable; ≥0.80 good; ≥0.90 excellent), reported together with 95% confidence intervals (CIs). Test–retest reproducibility was estimated by the intraclass correlation coefficient using a two-way mixed-effects model, absolute agreement and single-measure definition (ICC[A,1]), also reported with 95% CIs. Construct validity was explored conceptually through the predefined two-dimension structure and the pattern of patient responses. A two-sided *p*-value < 0.05 was considered statistically significant. Analyses were performed with IBM SPSS Statistics v.27 (IBM Corp., Armonk, NY, USA).

## 3. Results

The preliminary questionnaire was administered to 25 patients in the pilot phase in order to assess item comprehension and estimate internal consistency and reliability. The pilot patients had an average age of 73 ± 8 years, were roughly balanced by sex (14 men and 11 women), and received an average of 22 ± 2 h of treatment per day. Cognitive interviews confirmed adequate comprehension of the items, and the questionnaire was therefore finalised as shown in Supplementary Material File S1.

Internal consistency was good-to-excellent across both dimensions and the total scale, and test–retest reproducibility was excellent (Table 1).

The final cohort comprised 120 patients (62 men, 51.6%; 58 women, 48.4%), with a mean age of 78 ± 13 years (range 40–99) and a mean duration of LTOT of 40 ± 32 months. Demographic and clinical characteristics are summarised in Table 2. Although spirometric and gasometric severity variables were not available for all participants, the cohort represents routine LTOT practice in chronic hypoxaemic respiratory failure, with long exposure to home oxygen therapy (mean 40 ± 32 months).

Considering the sum of “somewhat”, “quite a lot” and “a lot” as a favourable response, perceived benefit was reported as follows: easier performance of daily efforts 68%, increased physical capacity 52%, reduction in dyspnoea 66%, improvement in general health 66%, improved self-confidence 67%, improved social life 57%, positive change in mood 57%, more enthusiasm to face the day 57%, and less effort in daily life 50% (Table 3).

Defining the favourable outcome as the sum of “nothing” or “very little”, 93% of patients had not required psychological support, 91% did not find the equipment uncomfortable at home, and 93% had not abandoned therapy due to nasal dryness or rhinitis. Conversely, 41% expressed concern about becoming dependent on oxygen, 44% considered the therapy an inconvenience when sleeping away from home, and 49% reported morning airway dryness (Table 3).

Among patients using ambulatory oxygen systems, the specific mobility item (2b) was retained as a descriptive question and excluded from the total score.

## 4. Discussion

This study describes the development, validation and field application of a brief PROM specifically designed for patients on chronic home oxygen therapy. The LTOT-PROM was structured to capture the patient-perceived impact attributable to LTOT during the previous 4 weeks in chronic users and showed good internal consistency together with excellent test–retest reproducibility, comparing favourably with previously published, more complex respiratory questionnaires [30,31].

Three findings deserve to be highlighted. First, a substantial proportion of patients perceived a clear therapeutic benefit in daily life, particularly in relation to less effort, less dyspnoea, better general health and improved self-confidence, which is consistent with the improvement in health-related quality of life described in patients appropriately treated with LTOT [9]. Second, although the treatment was generally well tolerated, clinically relevant adverse effects remained frequent, especially early-morning airway dryness, concern about dependence and inconvenience when sleeping away from home. These aspects are highly relevant in routine care because they represent precisely the kind of treatment burden that generic respiratory questionnaires often underrepresent [21,22,23,30,31]. Third, the two-dimensional structure allowed the instrument to distinguish between perceived benefit and treatment-related burden, providing a concise patient-centred profile that can be reviewed rapidly during outpatient follow-up.

Compared with the only previous Spanish PROM/PREM developed for the broader field of home respiratory therapies [31], our instrument is shorter, oxygen-specific and structured in two clearly interpretable dimensions, allowing for it to be used in busy outpatient clinics in less than three minutes. The qualitative concerns previously described by Caneiras et al. [22] and Clèries et al. [23]—social stigma, fear of dependence, perceived inconvenience and discomfort related to oxygen therapy—are explicitly captured by the items of the LTOT-PROM, which strengthens its content validity.

A relevant methodological clarification is that the LTOT-PROM was conceived as an attribution-based PROM with a standardised 4-week recall period, not as a pure responsiveness instrument requiring clinical change inside that month. Therefore, the wording “have you noticed that…” seeks to quantify the extent to which patients experience LTOT-related benefit or burden in their current daily life during the recall window. Under this interpretation, a clinically stable patient who continues to perceive less dyspnoea or easier daily efforts because of LTOT is still expected to respond in the favourable range; the instrument does not penalise stability when that stability is experienced as sustained benefit. Formal responsiveness to change should be addressed in future longitudinal validation studies.

The clinical applicability of this PROM is twofold. First, it offers the clinician a structured measure of patient-perceived benefit and burden that can complement physiological assessment and support shared decision-making [26,27]. Second, it can help identify specific practical problems amenable to intervention, such as troublesome dryness, equipment discomfort or mobility-related limitations, thereby facilitating a more individualised approach to LTOT follow-up. It also provides a pragmatic framework for discussing sustained benefit versus treatment burden in patients who are clinically stable but chronically dependent on oxygen support.

The possibility of administering the LTOT-PROM through a mobile application is also clinically attractive. Because the questionnaire is short, uses a simple Likert structure and focuses on experiences that patients can self-report easily, electronic administration could facilitate remote monitoring and longitudinal follow-up. Before routine implementation, however, equivalence between paper and digital administration, usability in older oxygen-dependent populations and data-protection requirements should be evaluated formally.

### Limitations

This is a single-centre study, which may limit generalisability. The pilot reliability sample (n = 25) is in the lower range of recommendations for PROM validation, although it provided consistent estimates of internal consistency and reproducibility. The present study focused on content development, cognitive testing and preliminary psychometric performance; further work should evaluate structural validity, external validity against established instruments, responsiveness to change and the relationship between LTOT-PROM scores and objective adherence measures. In addition, some clinically relevant descriptors of the cohort, including detailed physiological severity, diagnostic subgroup analyses and ambulatory-system subgroup reporting, were not developed in depth in this initial study. The high proportion of favourable responses in some adverse-effect items also suggests the possibility of ceiling effects that should be explored in future validation cohorts. In addition, we did not systematically collect physiological severity descriptors for all patients, which limits phenotypic granularity and subgroup interpretation. Nevertheless, all included participants had an established indication for home oxygen therapy and a prolonged mean exposure to LTOT, supporting the relevance of the cohort to real-world chronic hypoxaemic respiratory failure. Future multicentre validation studies should stratify LTOT-PROM results by diagnosis and objective severity markers.

## 5. Conclusions

We developed a brief, oxygen-specific PROM with good reliability and excellent reproducibility, applicable in routine clinical practice. The instrument captures both perceived therapeutic benefit and adverse effects in chronic home oxygen therapy and provides a concise patient-centred assessment of recent LTOT experience. Further studies should complete structural and external validation and determine its relationship with objective adherence outcomes The instrument is intended to capture patient-perceived benefit and burden attributable to LTOT during a standardised 4-week recall window, which may facilitate more precise interpretation in routine follow-up.

## Figures and Tables

**Table 1:** Reliability and reproducibility of the LTOT-PROM (pilot phase, n = 25).

Dimension	Items	Cronbach’s α (95% CI)	Interpretation	ICC (95% CI)	Interpretation
**Daily Activity**	9	0.814 (0.681–0.906)	Good	0.979 (0.953–0.991)	Excellent
**Adverse Effects**	6	0.743 (0.548–0.872)	Acceptable	0.874 (0.735–0.942)	Excellent
**Total scale**	**15**	**0.808 (0.677–0.902)**	**Good**	**0.890 (0.767–0.950)**	**Excellent**

ICC: intraclass correlation coefficient. CI: Confidence interval.

**Table 2:** Demographic and clinical characteristics of the field-application cohort (n = 120).

Variable	Value
**Age, years (mean ± SD; range)**	78 ± 13 (40–99)
**Sex, men/women, n (%)**	62 (51.6)/58 (48.4)
**Duration of LTOT, months (mean ± SD)**	40 ± 32
**Main indication**	COPD (>65% of LTOT prescriptions)
**Type of LTOT**	Stationary and ambulatory (mobile)
**Severity context of the cohort**	Routine clinical LTOT indication for chronic hypoxaemic respiratory failure; mean LTOT duration 40 ± 32 months.

LTOT, long-term oxygen therapy; SD, standard deviation; COPD, chronic obstructive pulmonary disease.

**Table 3:** Patient-reported responses to the LTOT-PROM (n = 120).

***Dimension 1. Daily Activity—favourable response = "somewhat/quite a lot/a lot"***		
**#**	**Item (“In the Last 4 Weeks, Have You Noticed That…”)**	**Favourable Response (%)**
**1**	…daily efforts are easier to perform?	68
**2**	…your physical capacity has increased?	52
**3**	…the feeling of shortness of breath has decreased?	66
**4**	…your general health has improved?	66
**5**	…your self-confidence has improved?	67
**6**	…your social life has improved?	57
**7**	…positive changes in your mood?	57
**8**	…you face the day with more enthusiasm?	57
**9**	…daily tasks require less effort?	50
***Dimension 2. Adverse Effects—favourable response = “nothing/very little”***		
**#**	**Item (“In the last 4 weeks…”)**	**Favourable response (%)**
**10**	…have you needed psychological support?	93
**11**	…have you been worried about becoming dependent on oxygen therapy?	59
**12**	…has the treatment been an inconvenience for sleeping away from home?	56
**13**	…have you felt the home oxygen equipment to be uncomfortable?	91
**14**	…have you noticed airway dryness in the early morning?	51
**15**	…have you stopped using the equipment due to nasal dryness or rhinitis episodes?	93
***Specific item—mobile oxygen only***		
**#**	**Item**	**Favourable response**
**2b**	Has your mobility improved?	Positive in the majority of mobile-LTOT patients

## Data Availability

The data presented in this study are available on request from the corresponding author. The data are not publicly available due to privacy and ethical restrictions.

## Supplementary Materials

The following supporting information can be downloaded at: https://www.mdpi.com/article/10.3390/jcm15134948/s1, Supplementary File S1. Final LTOT-PROM Questionnaire (definitive version, ready for professional translation).
