# Supplementary material for: Development and Application of Patient-Reported Outcome Measures (PROMs) in Patients on Chronic Home Oxygen Therapy
Source: J Clin Med. 2026 Jun 25;15(13):4948. doi: 10.3390/jcm15134948 (PMC13361745; doi:10.3390/jcm15134948)
Supplement: Supplementary file 1 [file jcm-15-04948-s001.zip › jcm-4367172-supplementary.pdf]

Supplementary File S1

Final LTOT-PROM Questionnaire (definitive version, ready for professional translation)

Reference manuscript: Chiner E, Boira I, Fernández-Serrano J, Llombart M, Fernández M, Gigliarano F, García-Ferrer S. "Development and Application of Patient-Reported Outcome Measures (PROMs) in Patients on Chronic Home Oxygen Therapy". Journal of Clinical Medicine, 2026.

CEIm approval code: 23/075 — Hospital Universitario San Juan de Alicante (Spain).

Instructions for the patient

This brief questionnaire (≈3 minutes) is part of your clinical follow-up. Please answer thinking about HOW YOU HAVE FELT DURING THE LAST 4 WEEKS while you have been receiving home oxygen therapy. For each item, please tick (✓) the option that best describes your experience. There are no right or wrong answers; only your own perception is relevant.

Response scale (5-point Likert)

0	1	2	3	4
**Nothing**	Very little	Somewhat	Quite a lot	A lot

Patient identification (to be completed by clinical staff)

Patient ID	Date (dd/mm/yyyy)	Type of LTOT	Months on therapy
______________	_____ / _____ / _________	☐ Stationary ☐ Ambulatory	______________

Dimension 1. Daily Activity

"In the last 4 weeks, have you noticed that…"

#	Item	0 Nothing	1 Very little	2 Somewhat	3 Quite a lot	4 A lot
1	…it is easier for you to perform daily efforts?	☐	☐	☐	☐	☐
2	…your physical capacity has increased?	☐	☐	☐	☐	☐
3	…the feeling of shortness of breath has decreased?	☐	☐	☐	☐	☐
4	…your general health has improved?	☐	☐	☐	☐	☐
5	…your self-confidence has improved?	☐	☐	☐	☐	☐
6	…your social life has improved?	☐	☐	☐	☐	☐
7	…there have been positive changes in your mood?	☐	☐	☐	☐	☐
8	…you face the day with more enthusiasm?	☐	☐	☐	☐	☐

9	…daily tasks require less effort from you?	☐	☐	☐	☐	☐

Dimension 2. Adverse Effects

"In the last 4 weeks…"

#	Item	0<br>Nothing	1<br>Very little	2<br>Somewhat	3<br>Quite a lot	4<br>A lot
10	…have you needed psychological support?	☐	☐	☐	☐	☐
11	…have you been worried about becoming dependent on oxygen therapy?	☐	☐	☐	☐	☐
12	…has the treatment been an inconvenience for sleeping away from home?	☐	☐	☐	☐	☐
13	…have you felt the home oxygen equipment to be uncomfortable?	☐	☐	☐	☐	☐
14	…have you noticed airway dryness in the early morning?	☐	☐	☐	☐	☐
15	…have you stopped using the equipment due to nasal dryness or rhinitis episodes?	☐	☐	☐	☐	☐

Specific item — patients on AMBULATORY (mobile) oxygen therapy only

"In the last 4 weeks, have you noticed that…"

#	Item	0<br>Nothing	1<br>Very little	2<br>Somewhat	3<br>Quite a lot	4<br>A lot
2b	…your mobility has improved?	☐	☐	☐	☐	☐

Scoring guidance

Each item is coded from 0 ("Nothing") to 4 ("A lot"). For Dimension 1 (Daily Activity) a favourable response is defined as "somewhat", "quite a lot" or "a lot" (codes 2–4). For Dimension 2 (Adverse Effects) the favourable outcome corresponds to absence of the adverse event, i.e. "nothing" or "very little" (codes 0–1). The total score may be reported by dimension and/or as a global summary. The mobile-LTOT item (2b) is analysed only in the sub-group on ambulatory oxygen and is not included in the total score.

Notes for professional translators

- The questionnaire was originally developed in Spanish and back-translated to English. Cross-cultural adaptation should follow the ISPOR Principles of Good Practice for the Translation and Cultural Adaptation Process for Patient-Reported Outcomes (PRO) Measures.
- Preserve the time frame "last 4 weeks" in all target languages.
- Maintain the 5-point Likert structure: Nothing / Very little / Somewhat / Quite a lot / A lot.
- Keep dimensions and item numbering identical to the source instrument.
- The mobile-LTOT item is conceptually linked to item 2 (physical capacity) and is therefore labelled "2b".
